# Reformers

**Published:** 1863-04

**Authors:** 


### REFORMERS.

The world is full of them, and yet the world is not reformed. Almost every man can tell you how things ought to be arranged so as to usher in the millenium, when everybody will be happy and good. But to make a theory available, it must be practicable, it must be a possibility. This very day, three men, a physician, a clergyman, and an editor, have propounded plans in our office for the amelioration of the condition of mankind.

The physician is aiming to get rich enough to supply all the poor with food and clothing at the actual cost of production.

The clergyman thinks that no radical progress can be made, until the government ceases to carry the mail on Sundays, and every man, woman, and child becomes a literal tee-totaller, and anti-slavery.

The editor believes that the secret of universal happiness is universal love; that everybody must love every other body as well as he loves himself, and that the rich in money should give their surplus to the rich in love and leisure, who should go about and find out the objects upon which to bestow it with a wise discrimination.

Our own plan differs from all these. It runs thus. The world can never be as happy as it ought to be, until all are Christians; the great instrumentality for effecting this, is the study and practice of what is contained in the Scriptures of the Old and New Testaments. The Bible is the sword of the Spirit, the instrument of the world's conversion. Whatever, then, promotes the reading and study of the Bible, promotes the world's redemption from sorrow and crime, its elevation to holiness and happiness.

To create a love for the reading and study of the Bible with the greatest certainty of success, we must begin early, long before a child can learn its alphabet; begin just as soon as an infant is observed to take an interest in any story, and then feed it daily with Bible stories, and incidents, and facts, all along creating a feeling of the most implicit reliance on the absolute truthfulness of every sentiment, sentence, and syllable; that these are truths coming from the lips of the loving Father of us all, whose desire is that every child of his should come eventually to his Father's house, to spend an eternity with him in Paradise, according to the Saviour's announcement, "that where I am, there ye may be also."

While all this is going on, facts should be thrown in from time to time, strongly fortifying the faith of the child in the literal accuracy of every Bible statement, how its histories are corroborated by profane histories, by the revelations of after events, and by the researches of science. Pains should be taken to collect as many explanations, and corroborations, and verifications of Bible narratives as possible, like the one on page fifty-five of the June number. From time to time, the testimonies of the great and good of all ages, as on page eighty-one of July, as to the value of the sacred Scriptures, should be presented; and these things should be continued until the child passes from under the parental roof and parental control. In the meanwhile however, begin quite as soon as the child is capable of understanding principles and abstract truths, to instill into its mind the distinctive doctrines of the church to which the parents belong, and thus they will become rooted and grounded in the faith of their fathers, will very uniformly grow up to be members of a particular sect from principle; and it needs no argument to prove that a man is an efficient member of a particular church in proportion as he is enthusiastic, theoretically and practically, as to its distinctive tenets. The milk and water folk who think that one church is about as good as another, that their own in particular is no better than any other, are not only of no use, they are a positive injury to any society, are a weight on its influence, a clog to its progress. To happify mankind, then, the most efficient method and the shortest in the long run, is to begin with our children while they are yet infants, and patiently and sedulously and prayerfully inculcate the sentiment that the Bible is the only safe rule of faith and practice, the sure and only sure guide to a blessed immortality.

